# Upcycling Real Waste Mixed Lithium-Ion Batteries by Simultaneous Production of rGO and Lithium-Manganese-Rich Cathode Material

**DOI:** 10.1021/acssuschemeng.1c04690

**Published:** 2021-09-24

**Authors:** Pier Giorgio Schiavi, Robertino Zanoni, Mario Branchi, Camilla Marcucci, Corrado Zamparelli, Pietro Altimari, Maria Assunta Navarra, Francesca Pagnanelli

**Affiliations:** Department of Chemistry, Sapienza University of Rome, Piazzale Aldo Moro n.5, 00185, Rome, Italy

**Keywords:** Lithium-ion battery recycling, Reduced graphene oxide, Lithium-manganese-rich cathode, Doped LMR, Hummers’ method

## Abstract

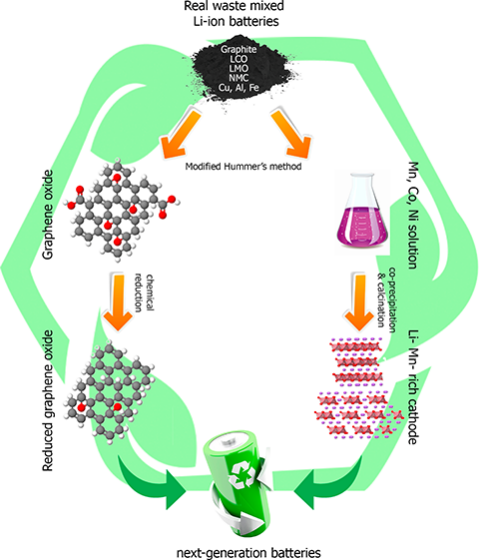

The direct synthesis of high-value products from end-of-life Li-ion batteries (LIBs), avoiding the complex and costly separation of the different elements, can be reached through a competitive recycling strategy. Here, we propose the simultaneous synthesis of reduced graphene oxide (rGO) and lithium-manganese-rich (Li_1.2_Mn_0.55_Ni_0.15_Co_0.1_O_2_ - LMR) cathode material from end-of-life LIBs. The electrode powder recovered after LIBs mechanical pretreatment was directly subjected to the Hummers’ method. This way, quantitative extraction of the target metals (Co, Ni, Mn) and oxidation of graphite to graphene oxide (GO) were simultaneously achieved, and a Mn-rich metal solution resulted after GO filtration, owing to the use of KMnO_4_ as an oxidizing agent. This solution, which would routinely constitute a heavy-metal liquid waste, was directly employed for the synthesis of Li_1.2_Mn_0.55_Ni_0.15_Co_0.1_O_2_ cathode material. XPS measurements demonstrate the presence in the synthesized LMR of Cu^2+^, SO_4_^2–^, and SiO_4_^4–^ impurities, which were previously proposed as effective doping species and can thus explain the improved electrochemical performance of recovered LMR. The GO recovered by filtration was reduced to rGO by using ascorbic acid. To evaluate the role of graphite lithiation/delithiation during battery cycling on rGO production, the implemented synthesis procedure was replicated starting from commercial graphite and from the graphite recovered by a consolidated acidic–reductive leaching procedure for metals extraction. Raman and XPS analysis disclosed that cyclic lithiation/delithiation of graphite during battery life cycle facilitates the graphite exfoliation and thus significantly increases conversion to rGO.

## Abstract 2

Starting from the electrodic powder of end-of-life Li-ion batteries, lithium- manganese-rich cathode material and reduced graphene oxide were obtained.

## Introduction

An unprecedented increase in the volume of end-of-life (EoL) lithium-ion batteries (LIBs) is expected over the next few years driven by the green energy transition. Particularly, the diffusion of electric vehicles can contribute to raise the volume of end-of-life LIBs. In this scenario, the recycling of EoL LIBs is fundamental not only to prevent the dispersion into the environments of toxic and/or harmful battery components but, particularly, to recover the strategic and critical raw materials (graphite, Co, Ni, Mn, Li, Cu, Al) to be reintroduced in the LIBs manufactory chain.^1,2^ Currently, the approach commonly followed to treat EoL LIBs includes a pyrometallurgical process where metals are smelted at high temperature with the loss of graphite, lithium, and manganese. In addition, by this process, metals are generally recovered as alloys and need to be further processed to obtain battery-grade materials.^3^ Hydrometallurgical recycling processes could configure a promising recycling alternative. These can allow recovering any battery material and are characterized by an environmental impact much lower than pyrometallurgical processes. However, hydrometallurgical processes are typically complex, including a lengthy sequence of process stages to separate the different battery materials, include the consumption of chemicals, and generate large solid and liquid waste volumes.^4^ An effective strategy to overcome these limitations is the “resynthesis” of battery materials.^5−7^ This strategy includes directing the hydrometallurgical process toward the resynthesis of battery materials, avoiding the complex and costly separation of the different metals. Over the past years, several studies were reported demonstrating the potential of hydrometallurgical processes to recover all of the materials of interest as directly reusable battery materials. Particularly, this strategy enables direct production of a metal–lithium mixed oxide material, such as LiMn_*a*_Ni_*b*_Co_*c*_O_2_ (NMCs), without the need to separate the different metals as high-purity salts.^5−7^ However, it is well-known that the low capacity of NMCs and of the other currently available cathode materials (LiCoO_2_, NCA, LiFePO_4_, LiMn_2_O_4_) (ranging from 100 to 180 mAh g^–1^) represents the bottleneck of LIB performances.^8^ In addition, most of the performing cathode materials have been traditionally cobalt-based, which has determined an intensive exploitation of primary cobalt sources and an increase in the cobalt price.^9^

For these reasons, efforts are currently devoted to the development of new high-performance cathode materials with a lower cobalt content. In this framework, particular attention has been attracted by lithium- and manganese-rich oxides (LMR) due to their high capacity (exceeding 250 mAh g^–1^)_,_ operative discharge voltage higher than 3.5 V, and lower costs due to the low cobalt content.^10^ Nevertheless, the activation and phase transition of LMR from a layer to a cubic spinel-like phase determine unsatisfactory capacity retention and severe voltage decay, which can hinder commercial application.^8^ To solve these drawbacks, several strategies have been developed to improve LMR stability, including a different synthesis method,^11^ coating,^12^ blending,^13^ and ionic doping.^14,15^ Among these strategies, anionic doping using polyanions of nonmetal elements, such as PO_4_^3–^,^16^ SO_4_^2–^, and SiO_4_^4–^, has been demonstrated to greatly improve the cycling durability and voltage fading.^17^ In accordance with this analysis, the sustainability of hydrometallurgical resynthesis processes could be further increased by addressing the hydrometallurgical recycling of LIBs toward the direct synthesis of a Li- and Mn-rich material, either by processing LIB feedstocks with larger Mn content or by the addition of Mn to the solution generated by electrode powder leaching. A synergy strategy that can be followed to increase the competitiveness of the LIBs recycling process is the simultaneous recovery of the anode graphite fraction. This fraction has been rarely reported to be recovered and tested for reuse in the study of hydrometallurgical processes.^18^ Despite this apparent lack of interest toward graphite recovery, carbon-based nanomaterials, such as carbon fibers, carbon nanotubes, nanoribbons, graphene, and reduced graphene oxide, have attracted considerable attention as battery electrode materials.^19,20^ Particularly, graphene is considered to represent the candidate for next-generation energy storage devices, though it has a considerably wider application range. In this framework, chemical oxidation of graphite to graphene oxide (GO) and its subsequent reduction configures the simpler scalable method to synthesize graphene, or, more precisely, reduced graphene oxide (rGO). To date, many methods have been developed to synthesize GO, among which the most widely adopted are the Hummers’ method and its modified formulations.^21^ The Hummers’ method includes use of KMnO_4_ as an oxidizing agent and a low solid-to-liquid ratio, i.e., a low ratio between the graphite mass and the reactant solution volume. This nevertheless produces a huge liquid volume containing manganese, which represents a waste to be treated. In addition, the Hummers’ method is generally applied to synthetic graphite or preoxidized and purified graphite, which ultimately makes the process scarcely economical.^22^

Here, we propose an innovative process to directly synthesize a layered LMR and rGO from EoL LIBs. The proposed recycling strategy relies on the application of a modified Hummers’ method to process the electrodic powder delivered by pilot-scale mechanical pretreatment of mixed Li-ion batteries. The modified Hummers’ method has allowed for the quantitative extraction of metals from the electrodic powder and the production of GO without any metal impurity. The resulting solution contains the metals from the cathode materials and a significant amount of manganese from the KMnO_4_ used in the Hummers’ method. From such a consideration derive the idea to synthesize LMR cathode material keeping cobalt in defect and adding only Mn and Ni to correct the stoichiometry of metals to yield Li_1.2_Mn_0.55_Ni_0.15_Co_0.1_O_2_. The electrochemical performances of the produced cathode materials are thoroughly analyzed and compared with literature data for the same LMR produced using commercial reagents. The effect of graphite lithiation/delithiation during battery cycling on the production of graphene oxide is evaluated.

## Materials and Methods

### Electrodic Powder

Exhausted lithium-ion batteries were collected and crushed by SEVal Group s.r.l., an Italian waste disposal company. The electrodic powder was obtained by sieving the crushed material with a vibrant sieve with a grid mesh size of 0.5 mm. The metal content in the electrodic powder was estimated by microwave-assisted digestion in aqua regia of six samples of 0.5 g (Milestone Ethos 900 Microwave Labstation). The quantitative determination of metals was carried out by atomic absorption spectrophotometer (AAS - ContraAA 300 - Analytik Jena AG).

### Metals Extraction—Synthesis of rGO

Metals extraction from the electrodic powder was done using the revised Hummers’ method,^23^ where NaNO_3_ is not employed as an intercalator agent.^24^ The revised Hummers’ method involves the use of concentrated H_2_SO_4_ and KMnO_4_ both as an intercalator and an oxidizing agent and H_2_O_2_ (30% w/w) as a reducing agent. This treatment was directly applied to the electrodic powder. Reagent quantities for the synthesis of graphene oxide from graphite by the Hummers’ method were adapted to the electrodic powder considering its graphite content. In particular, the solid to liquid ratio between graphite and H_2_SO_4_ is 1:24, the ratio between graphite and KMnO_4_ is 1:3, and that between graphite and H_2_O_2_ is 1:5. Electrode powder and H_2_SO_4_ were mixed in a jacket reactor under magnetic stirring. After, the reactor was placed in an ice bath and KMnO_4_ was slowly added avoiding the temperature increases above 20 °C. The temperature was then increased to 40 °C and held for 30 min. Subsequently, distilled water was added, and reaction temperature was kept at 95 °C for 30 min. Finally, H_2_O_2_ (30% w/w) was added and reaction proceed for the last 30 min.

The above described procedure produces, after filtration, a solid composed by graphite oxide and a solution containing the extracted metals and the Mn added as KMnO_4_. Graphite oxide was exfoliated by ultrasonication (Elmasonic S) for 30 min, and afterward, it underwent centrifugation at 3000 rpm for 40 min to remove nonexfoliated graphite.^23^ The obtained graphene oxide was dried and then reduced by chemical reduction employing l-ascorbic acid, under alkaline conditions.^25^ The reaction was carried out in an aqueous environment. The solid to liquid ratio between graphene oxide and H_2_O is 1:10000, and the ratio between graphene oxide and ascorbic acid is 1:10. As an alkaline agent, NH_4_OH (28–30%) was used and it was added until pH 9–10 was reached. Alkaline conditions are decisive to support the electrostatic repulsion between the negatively charged GO sheets.^25^ For comparison, rGO was also synthesized using the reported procedure but starting from a commercial graphite (Sigma-Aldrich 99.999%) and from a graphite recovered after two subsequent leaching treatments of the electrodic powder using H_2_SO_4_ and H_2_O_2_ to completely remove the metals (H_2_SO_4_ 1.5 M, solid/liquid 1:10, 85 °C, H_2_O_2_ 15% v/v, 3 h).^26^ The metal extraction percentages attained by applying the Hummers’ method and commonly used leaching treatment were calculated based on the following relationship
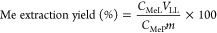
1where *C*_MeL_ is the metal concentration in the metal solutions (leach liquor) (mg L^–1^), *C*_MeP_ is the concentration of the metal Me in the electrodic powder (mg g^–1^), *V*_LL_ (L) is the volume of the leach liquor, and *m* is the amount of treated electrodic powder (g).

### Synthesis of Li_1.2_Mn_0.55_Ni_0.15_Co_0.10_O_2_ (LMR)

The manganese-rich metal solution coming from the treatment of the electrodic powder with the Hummers’ method was used as a source for the synthesis of the mixed metal hydroxide precursor of Li_1.2_Mn_0.55_Ni_0.15_Co_0.10_O_2_. The solution was first brought to pH 5.5 in order to selectively precipitate Fe, Cu, and Al impurities coming from metallic case and current collectors, respectively. The precursor synthesis was then based on a co-precipitation process using 0.1 L of purified solution in a nitrogen atmosphere and in the presence of NH_4_OH as a chelating agent.^27^ NaOH solution was added under vigorous stirring until pH 11 was reached. Cobalt is the metal with the lowest content in the LMR, and thus, it was kept in defect. Only NiSO_4_·6H_2_O and MnSO_4_·H_2_O were added (1.7 and 3.1 g, respectively) to ensure the stoichiometric ratio Mn/Ni/Co = 0.55:0.15:0.10. The precipitate was grounded in agate mortar with Li_2_CO_3_ as the lithium source that was added in 10% excess with respect to stoichiometry to compensate lithium evaporation during the calcination. Finally, the solid was calcined for 5 h at 450 °C and then the temperature was increased at 900 °C for 10 h.

### Chemical–Physical Characterization

Chemical, structural, and morphological characterization of graphene oxide (GO), reduced graphene oxide (rGO), and Li_1.2_Mn_0.55_Ni_0.15_Co_0.10_O_2_ was performed through Raman spectroscopy (Renishaw in Via spectrometer, laser source of argon ions 514 nm), scanning electron microscope (SEM, Zeiss Auriga), X-ray diffraction (XRD, Rigaku, D-Max Ultima), and X-ray photoelectron spectroscopy (XPS, modified Omicron NanoTechnology MXPS system). The XPS spectra were excited by achromatic Mg Ka photons (*h*ν = 1253.6 eV), generated operating the anode at 14 kV, 13 mA. Experimental spectra were theoretically reconstructed by fitting the peaks to symmetric Voigt functions and the background to a Shirley or a linear function. XPS atomic ratios (±10% associated error) were obtained from experimentally determined area ratios, corrected for the corresponding theoretical cross sections and for a square-root dependence of the photoelectron kinetic energies. All samples experienced charging under X-rays because of their mounting on insulating Teflon tape covering the tips, a procedure which was chosen in order to eliminate the presence of carbon and oxygen contribution coming from the tips in the useful C 1s and O 1s energy range. Quantitation with the C 1s peak was conducted after numerical removal by a software routine of the contribution given by Mg Ka_3_ and Mg Ka_4_ components. The binding energy scale was referenced to the C 1s position of the first peak component of each spectrum, obtained from reference measurements, run on conductive tips, typically falling at 284.2 eV. Care has been taken to produce curve fitting with closely matching fwhm and relative positions of the peaks.

### Electrochemical Characterization

Electrodes were constructed by mixing the active rGO or LMR materials, conductive carbon black (carbon Super-P, Timcal), and polyviniyidene fluoride (PVDF, Solvay 6020). The mixture was prepared as a slurry in *N*-methylpyrrolidinone (NMP) in a weight ratio of 80:10:10. LMR and rGO electrodes were obtained by the “doctor-blade” and drop casting methods, respectively. In both cases, the electrodes were vacuum-dried overnight at 140 °C. In order to evaluate the electrochemical performance of recovered rGO and LMR materials, different cell configurations were assembled and evaluated by galvanostatic cycling, rate capability, and cyclic voltammetry experiments. Each cell was assembled in an argon-filled glovebox with a content of O_2_ and H_2_O less than 1 ppm. A two-electrode R2032 coin-cell configuration using a lithium metal disk as the counter electrode and LP-30 (Solvionic 1.0 M LiPF_6_ in ethylene carbonate:diethyl carbonate 1:1 in volume) as the electrolyte was assembled for galvanostatic cycling and rate capability tests. C-rate experiments using rGO were carried out increasing the current after every 10 cycles from 100 to 800 mAh g^–1^ in a potential range between 0.02 and 2.8 V. Cycling performances of LMR material were investigated by galvanostatic cycling in a potential range between 2.5 and 4.7 V and with a constant current value corresponding to 0.1 C (1 C = 240 mAh g^–1^).^28^ For rate capability experiments, current values corresponding to 0.1, 0.2, 0.5, 1, and again 0.1 C were adopted. Finally, a three-electrode configuration “T-cell” using the active material as a working electrode and lithium both as a reference electrode and a counter electrode was assembled for cyclic voltammetry experiments. Cyclic voltammetry experiments were carried out between 2.5 and 4.7 V and with a scan rate of 0.1 mV s^–1^.

## Results and Discussion

**Figure 1:**
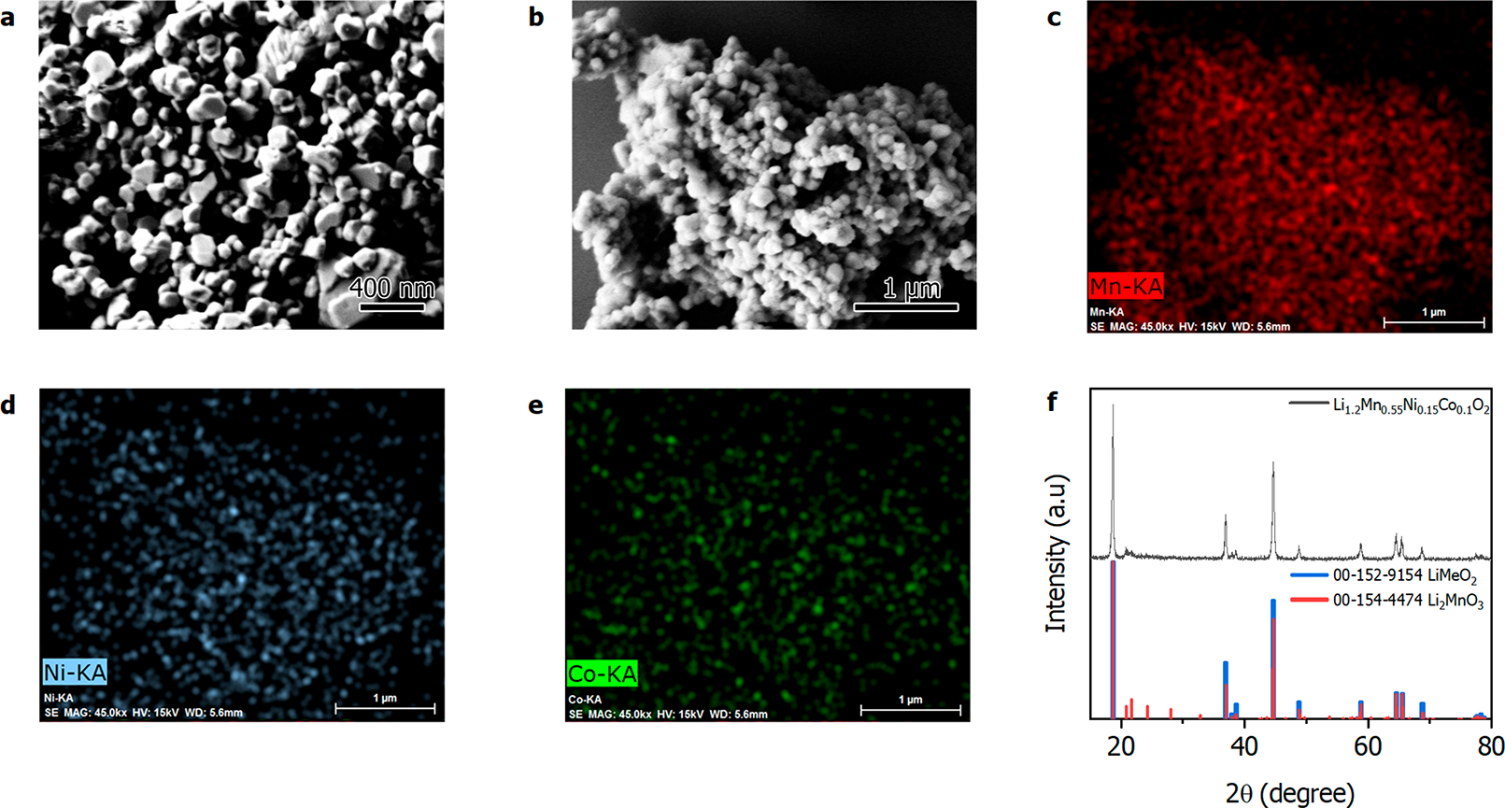
SEM images at different magnification (a, b) and EDX compositional maps (c–e) of the recycled LMR. (f) XRD pattern of the recycled LMR and reference pattern for the LiMeO_2_*R*3̅*m* and Li_2_MnO_3_*C*2/*m* space groups.

**Figure 2:**
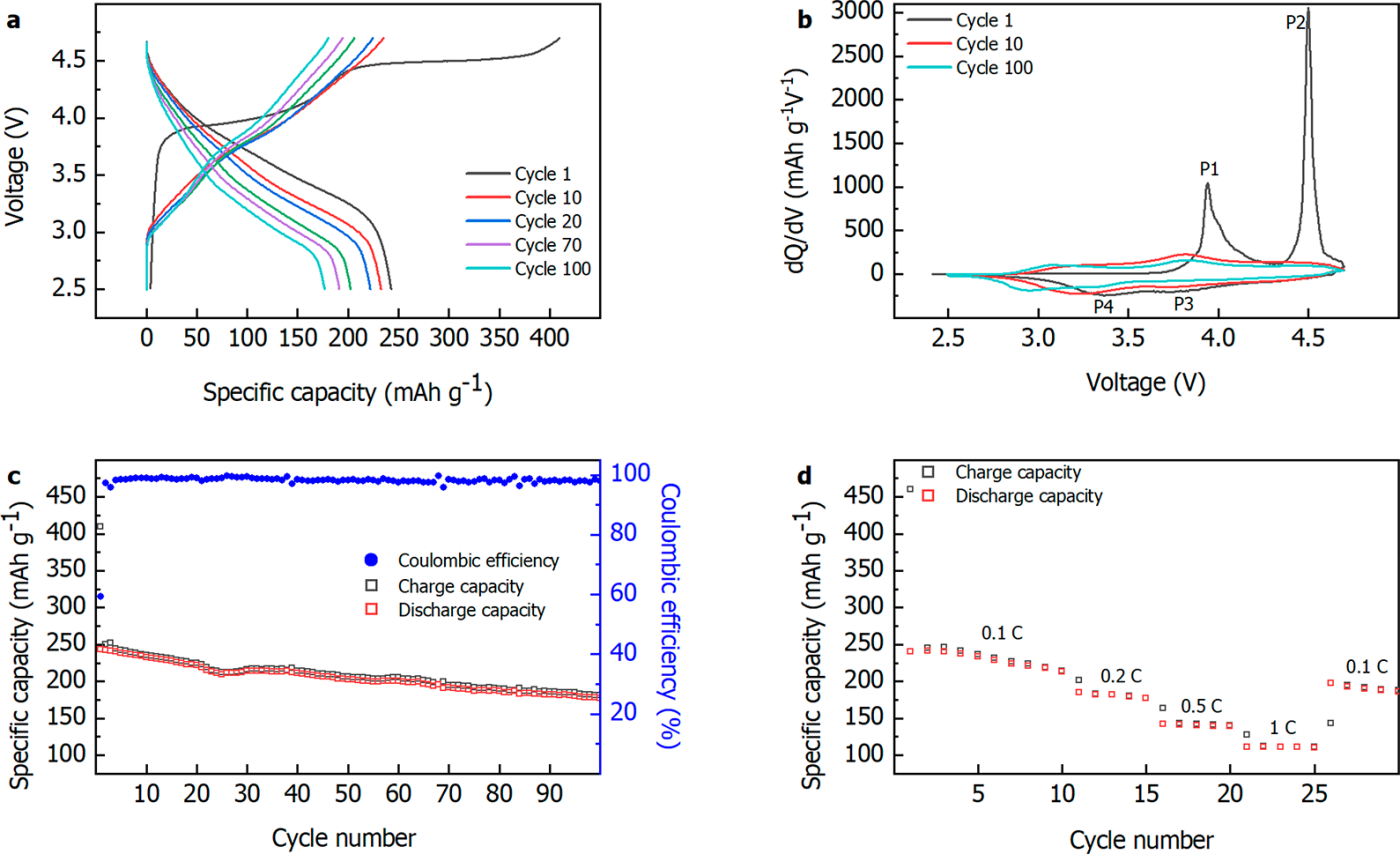
Recorded potential profiles during galvanostatic cycling (a), derivative capacity (b), galvanostatic cycling at 0.1 C (c), and rate capability (d) of Li_1.2_Ni_0.15_Mn_0.55_Co_0.1_O_2_ between 2.5 and 4.7 V.

Table 1 displays the metal content of the electrodic powder. It is apparent that cobalt is the metal with the highest concentration, which can be explained by the widespread application of lithium cobalt oxide batteries over the past years. Manganese and nickel are the other main metals, while Fe, Cu, and Al are present in lower concentration. The presence of these latter metals can be imputed to the metallic case and current collector fragments with dimensions lower than 0.5 mm generated during crushing and not removed during the subsequent physical dry separation (i.e., magnetic separation, sieving). The electrodic powder was employed as raw material for the production of graphene and LMR cathodic material. To this purpose, the Hummers’ method was directly applied to process the electrodic powder. The Hummers’ method involves the use of sulfuric acid and potassium permanganate, both as intercalating and oxidizing species, and hydrogen peroxide, as a reducing agent. Excluding potassium permanganate, sulfuric acid and hydrogen peroxide are generally used to perform acid-reductive leaching of LIBs electrodic powder with high extraction yields for the target metals (Co, Ni, Mn).^29^ The resulting solution obtained after the application of the Hummers’ method on the electrodic powder, that generally constitutes a liquid waste with high Mn concentration, was characterized by AAS and the solution composition (Hummers’ leachate) was reported in Table 1. As shown in Table 1, besides Mn added as KMnO_4_ in the application of the Hummers’ method, we also found all of the other metals contained in the electrodic powder. The Hummers’ leachate composition was used to compute the extraction yields of the metals. These values were benchmarked against the results attained using a consolidated acid-reducing leaching procedure for the extraction of metals from the electrodic powder.^26^Figure S1 in the Supporting Information displays that the Hummers’ method allows for a quantitative extraction of all of the metals and, in particular, the extraction yield for any target metal Co, Ni, and Mn is higher than that obtained with the conventional extraction procedure. Prior to using the Hummers’ leachate for the synthesis of the LMR cathode, a purification stage was performed by increasing the pH of the solution to remove Cu, Al, and Fe impurities. The resulting solution contains as main metal Mn. Due to increasing interest in the substitution or reduction of Co on the LIB active materials, Co was maintained in defect and only Mn and Ni as sulfate salts were added to correct the stoichiometric composition in the Hummers’ leachate. It should be noticed that most of the Mn that is found in the produced LMR, in addition to the Mn from the electrode powder, comes from KMnO_4_ used to produce GO. This latter Mn amount would be lost, thus becoming a waste, in the absence of subsequent direct synthesis of the LMR cathode material. Therefore, in addition to the Co, Ni, and Mn amounts from the electrode powder, the process allows effectively exploiting the Mn added as KMnO_4_, not only to perform GO production but also to sustain the production of the LMR material. After the precipitation, lithium carbonate was added with 10% excess respect the stoichiometry to the recovered solid mixed hydroxide and the mixture was calcined to obtain the respective oxide. SEM images (Figure 1a,b, Figure S2) of the synthesized LMR disclose a nanostructured oxide with nanoparticle dimensions of 80 ± 30 nm. EDX mapping (Figure 1c–e) of the LMR demonstrates that the Mn, Ni, and Co are homogeneously distributed in the powder, providing a first indication that a homogeneous crystalline phase was produced without the formation of segregated oxide phases. EDX also reveals (Table S1) the presence of Si (1.7 at. %) and S (0.2 at. %). LMRs can be considered as composite materials or solid solutions. Particularly, it is reported that the atomic structure of LMRs involves the coexistence of two crystalline phases, a trigonal phase with space group *R*3̅*m* typical for a mixed oxide LiMeO_2_ (Me = Ni, Mn, and Co) and a monoclinic phase with space group *C*2/*m* related to a Li_2_MnO_3_ oxide. LMR structures are composed of octahedral sites alternately filled by layers of LiMeO_2_ or Li_2_MnO_3_. In accordance with the described atomic structure, the recorded diffractogram (Figure 1f) of the recycled LMR shows the co-presence of the two crystalline phases contained in the composite LMR. The recovered LMR was used as a cathode material in a half lithium cell using metallic lithium as an anode. The LMR electrochemical characterizations show a behavior similar to that found for the LMR currently studied and obtained from synthetic reagents.^10−12^ Parts a and b of Figure 2 display the charge/discharge potential profile and the corresponding d*Q*/d*V* curves. In the first charge cycle, two potential windows characterized by large plateaus can be clearly identified. The first plateau detected over the range 3.9–4.4 V in Figure 2a, which corresponds to the peak at 3.9 V in Figure 2b (P1), can be attributed to the redox contribution of Ni^2+^/Ni^4+^ and Co^3+^/Co^4+^ associated with the insertion of lithium into the LiMeO_2_ layers of the composite LMR and can be described as follows:^10^
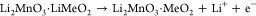
2

**Table 1:** Composition of Electrode Powder and Leachate Resulting after Treating the Electrode Powder with the Hummers’ Method

metal	powder composition (mg g^–1^)	Hummers’ leachate (mg L^–1^)
Co	183 ± 3	2714.9 ± 81.5
Mn	87 ± 1	6048.6 ± 102.8
Ni	51.7 ± 0.7	677.1 ± 6.7
Li	37.8 ± 0.3	629.5 ± 12.6
Fe	20.7 ± 0.2	200.3 ± 4.6
Cu	8.7 ± 0.6	138.3 ± 3.5
Al	2.90 ± 0.04	50.7 ± 3.0

The second plateau above 4.4 V in Figure 2a and the corresponding peak in Figure 2b (P2) can be attributed to the delithiation of Li_2_MnO_3_ accompanied by the evolution of oxygen after its extraction from the lattice of Li_2_MnO_3_. The mechanism is described by the following reaction:^10^
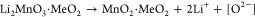
3

**Figure 3:**
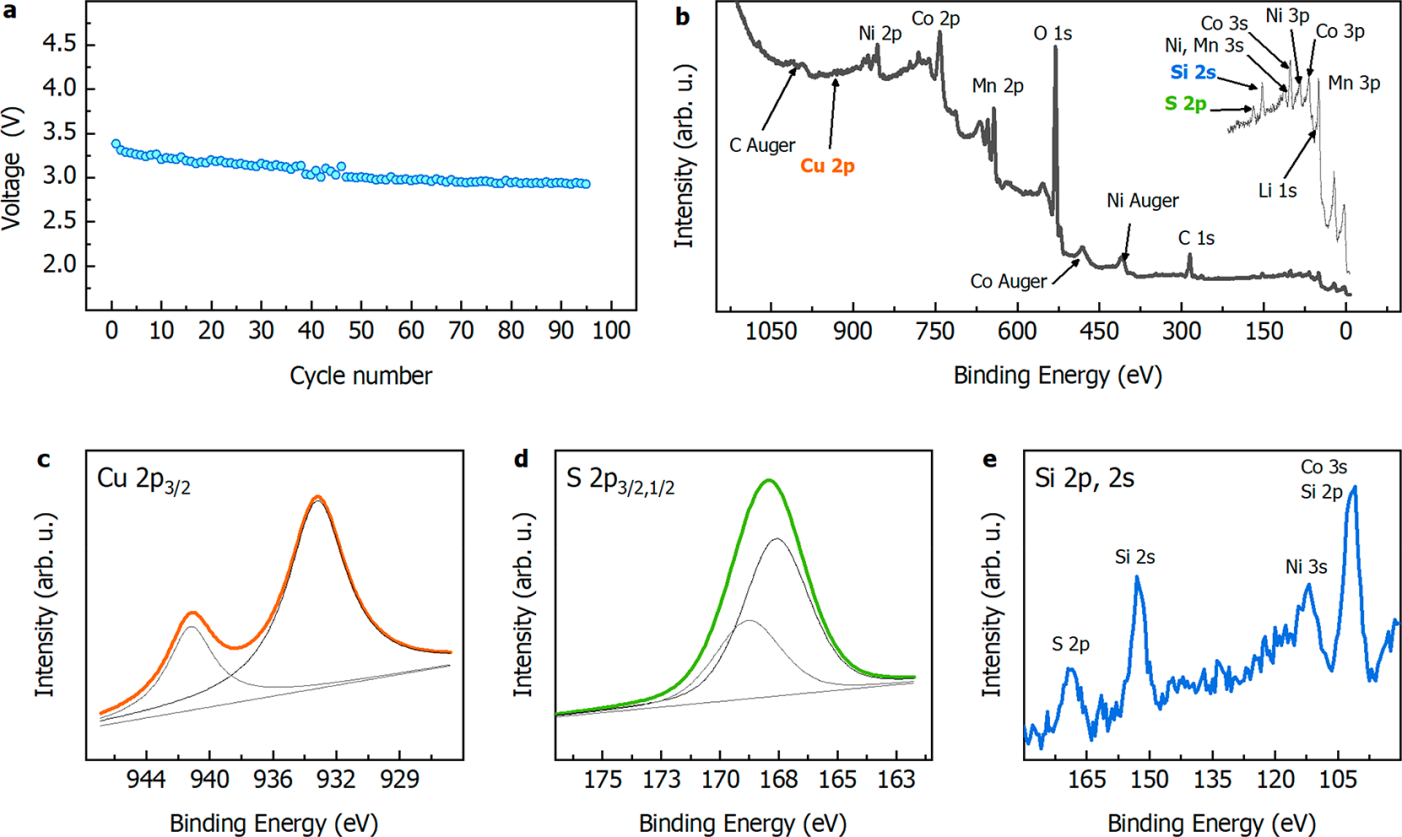
(a) Voltage decay per cycle of recovered LMR related to MnO_2_ lithiation. (b) XPS survey spectra or recycled LMR. (c–e) 2p spectra of Cu, S, and Si impurities, respectively.

**Figure 4:**
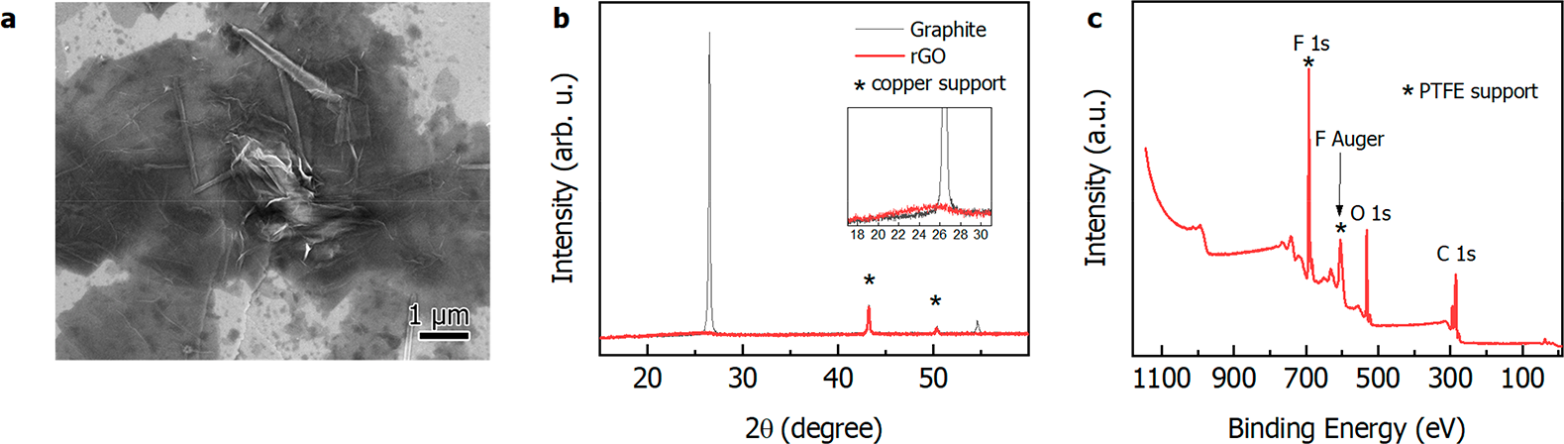
(a) SEM images of rGO nanoflakes (dark gray–black) onto silicon wafers (light gray), obtained by reducing the GO resulting after the application of the Hummers’ method directly on the electrodic powder. (b) XRD pattern for commercial graphite and rGO. (c) XPS survey spectra for the rGO sample.

**Figure 5:**
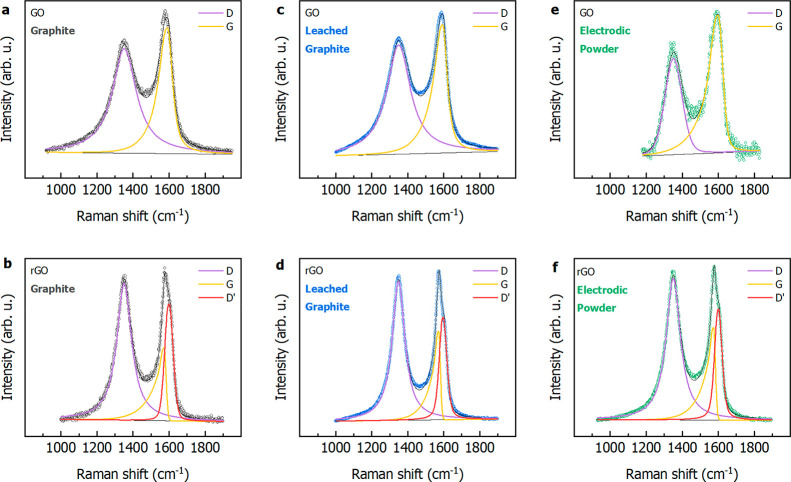
Raman spectra of the GO and rGO obtained applying the Hummers’ method on commercial graphite (a, b), graphite recovered from end-of-life LIBs by two subsequent acid leachings (c, d), and electrodic powder containing both end-of-life graphite and cathode materials.

**Figure 6:**
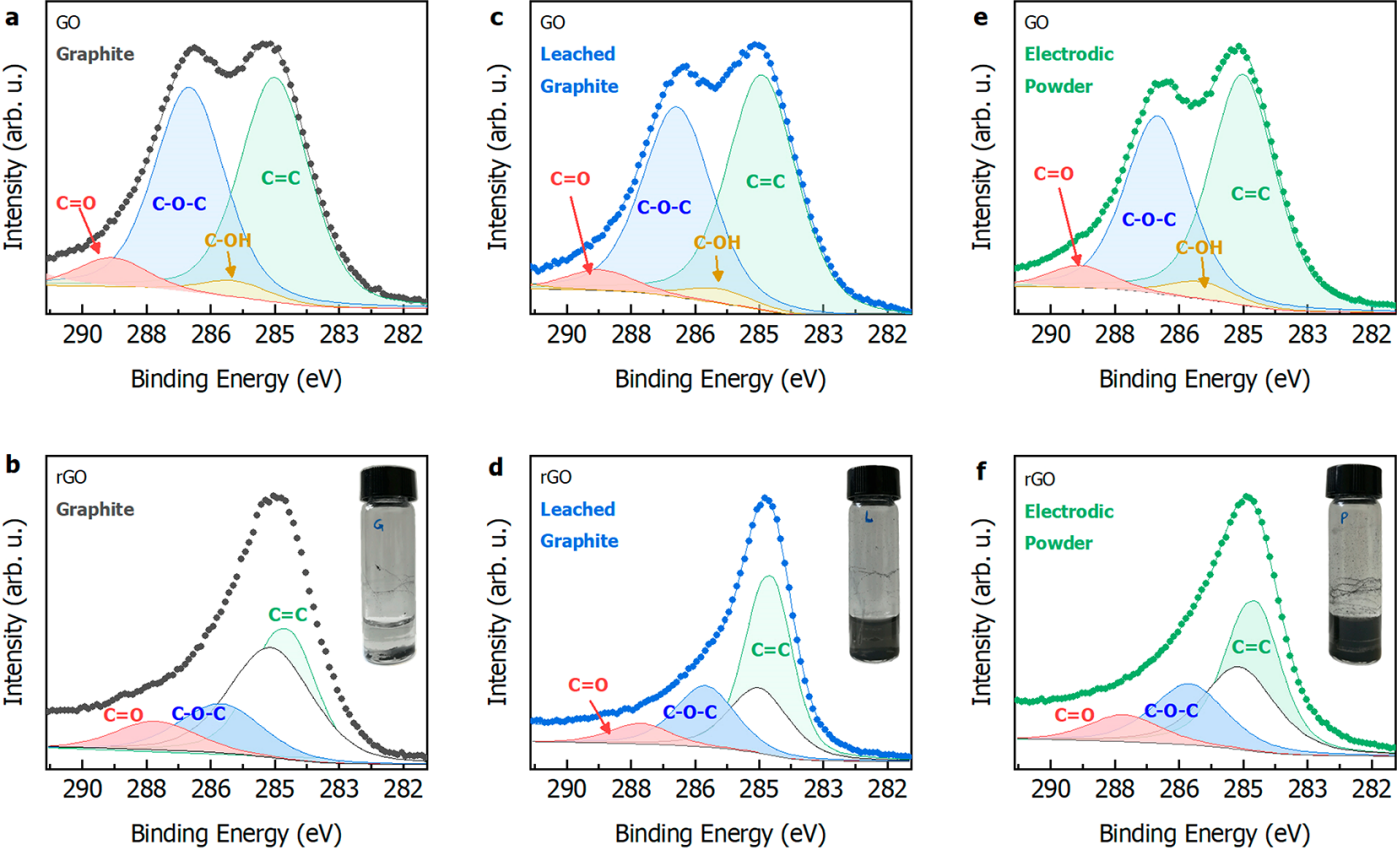
XPS C 1s spectra of the GO and rGO samples obtained applying the Hummers’ method on commercial graphite (a, b), graphite recovered from end-of-life LIBs by two subsequent acid leachings (c, d), and electrodic powder containing both end-of-life graphite and cathode materials. (b, d, f insets) The rGO suspensions on standing after 24 h.

Starting from the second charge, the potential profile differs from the first one, as a result of the activation process and irreversible structural changes of the LMR. The mean voltage decreases, which is clearly evidenced by the shift of the peaks toward lower values in the d*Q*/d*V* plot (Figure 2b). This phenomenon can be mainly attributed to the transformation of Li_2_MnO_3_ from layered to spinel structure, which currently hinders the practical application of LMRs. To evaluate the capacity retention of the recovered LMR, prolonged galvanostatic cycling was carried out (Figure 2c). A capacity on the second cycle of 242 mAh g^–1^ was found, which is very close to the theoretical value (251 mAh g^–1^). After 100 cycles, the capacity value decreases to 177 mAh g^–1^, corresponding to a capacity retention of 73%, which can be considered relatively high for a pristine LMR material.^10,15^Figure 2d exhibits the rate performance of recovered LMR at different rates. By increasing the current density, the discharge capacity decreased, reaching 110 mAh g^–1^ at 1 C. Figure 3a displays the voltage decay per cycle associated with lithiation of MnO_2_, formed in P3 (Figure 2b), to LiMnO_2_ (P4, Figure 2b). Starting from the second cycle, the potential associated with MnO_2_ lithiation shifts, after 100 cycles, downward from 3.3 to 2.9 V with a mean voltage decay per cycle of 4 mV. It should be emphasized here that a voltage decay of such magnitude is generally reached only after the improvement of LMR materials by optimizing the synthesis method, composition, morphology, doping, and surface coatings of LMR.^8,10^ Due to the complexity of the solution employed in this work for the synthesis of the LMR precursor, an in-depth characterization of the cathode materials was then performed to evaluate the co-presence of other metals or doping ions that may have enhanced both capacity retention and voltage decay of recycled LMR. To this purpose, LMR was dissolved and the attained solution was analyzed by AAS. This way, a Cu content of about 0.2 wt % was found. XPS survey spectra (Figure 3b) confirm the presence of Si and S, as revealed from EDX and the presence of Cu from AAS. Additionally, XPS quantitative analysis displays a surface enrichment for Cu and S, with a Cu content of about 4 at. % and about 20 at. % for sulfur. Other prevailing contributions from XPS survey spectra can be attributed to Mn, Co, Ni (2p, 3p, and 3s) and O (1s). Cobalt, manganese, and nickel 2p 1/2 and 2/3 binding energies and line shapes (Figures S4–S7) are close to those reported for LMR.^30^ To elucidate the contribution of the mentioned impurities, Cu, S, and Si 2p spectra (Figure 3c–e) were analyzed. The Cu 2p 3/2 peak (933.6 eV, Figure 3c) and the associated satellite can be assigned to the Cu(II) component, while S 2p and Si 2p and 2s spectra could be respectively referred to SO_4_^2–^ (102.5 eV, Figure 3d) and SiO_4_^4–^ (2p, 102.5 eV, Figure 3e). The presence of sulfate anion in LMR could be due to its high concentration in the solution employed for the synthesis of a mixed-hydroxide precursor, since concentrated sulfuric acid was used in the Hummers’ method and sulfates of Ni and Mn were added to correct the stoichiometry. Silicate can be due to the oxidation of silicon during precursor calcination. Silicon presence (<1 wt %) was declared in several LIB safety data sheets. Remarkably, doping LMR with Cu^2+^ has been reported to improve its cycling performance due to the better movement of Li ions into the enlarged unit cell, thus resulting in a reduction of charge-transfer resistance.^31^ It was also reported that SO_4_^2–^ and SiO_4_^4–^ polyanions, introduced into the LMR as doping agents, can change the layered structure and enhance the binding energy of cations to anions with the consequent inhibition of the metal migration during cycling.^17^ To further evaluate a possible doping effect of such impurities, Rietveld refinement of XRD spectra was carried out (Table S2). The *a* and *c* lattice parameters that we found are higher with respect to pristine LMR,^12^ implying the expansion of the Li layer in the layered structure due to the larger thermochemical radii of SO_4_^2–^ (258 pm) and SiO_4_^4–^ (240 pm) with respect to spherical O^2–^ (124 pm).^16^ Therefore, both copper and polyanion impurities that we found in the LMR, coming from the complex matrix that waste LIBs represent, have allowed for the improvement of the cycling performance of the recycled LMR. The solid resulting after the application of the Hummers’ method to process the waste electrodic powder was sonicated to produce GO and then reduced with ascorbic acid for the synthesis of rGO. Typical graphene nanoflakes were found during SEM analysis (Figure 4a, Figure S8), highlighting the losses of a well stacked graphite layer. Figure 4b displays XRD patterns for commercial graphite and rGO synthesized after the reduction of GO with ascorbic acid. The intense graphitic peak at 26.6° generally shifts to lower diffraction angle (∼10°) after interspacing distance changing as result of oxidation and intercalation of oxygen functional groups passing from graphite to GO. The disappearance of the graphitic peak in the rGO sample can be attributed to the exfoliation of layered structures of graphite oxide. The low intensity and broad peak at about 25° (Figure 4b, inset) could be due to a partial restacking of exfoliated graphene layers after the removal of oxygen functionalities and the consequent restoration of C=C bonds.^22,32^Figure 4c shows the XPS wide-range spectra for the rGO sample where, notably, no metals can be traced, confirming the quantitative extraction of metals reached applying the Hummers’ method. In order to figure out the role of the graphite lithiation/delithiation process during battery cycling in the production of rGO, GO and rGO were also synthesized starting from commercial graphite and from a graphite recovered from the same electrodic powder but by applying two subsequent acid-reductive leaching stages (H_2_SO_4_ + H_2_O_2_) to completely remove the metals. Raman spectra for GO and rGO samples from the three series of GO and rGO samples are collected in Figure 5. All of the spectra are reported with their associated theoretical curve fit results, conducted by means of symmetrical (D and D′) and skewed (G) curves, by employing the minimum number of components for a closer comparison among different samples. The spectra clearly show the differences expected in the sequence GO to rGO. These consist of the following: (i) an increase in the intensity ratio between the D and G bands, *I*_D_/*I*_G_, respectively falling at ∼1350 and ∼1600 cm^–1^; (ii) a narrowing of the D and G features, with the D′ peak emerging in all rGO samples from the G complex line shape; (iii) a sizable red shift in the G peak position. All of the above variations are experimentally confirmed for the three series, as can be seen in Table S2. The high-energy region is also affected in its position, shape, and width. An *I*_D_/*I*_G_ area ratio significantly higher for rGO than for the corresponding GO sample implies the formation of new domains of conjugated sp^2^ carbon atoms inside a network of both sp^3^ and sp^2^ carbons, following the partial removal of C—O groups. The shift of the G band, experienced by the full series of samples upon reduction of GO to rGO, can be attributed to a partial recovery of the hexagonal sp^2^ carbon network.^33^ The average crystallite domain size of the sp^2^ lattice results from the following relation^34^
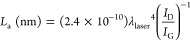
4amounting, in our case, to 13.61 × *I*_D_/*I*_G_. As can be seen in Table S2, the sample deriving from electrodic powder presents the smallest L_a_ in the series, both as GO and as rGO. The shape, width, and position of the 2D peak have been associated with the number of layers in few-layer graphene. In the series of investigated samples, a fwhm value of 100 cm^–1^ has been found for the commercial graphite and 130 cm^–1^ has been found for the other two, which translates into few-layer graphene in all cases, since a correspondence between 7 and 8 layers and 125 cm^–1^ has been given in the literature.^35^ The C 1s region of all of the GO samples (Figure 6a,c,e) presents close line shapes, with only minor variations in the relative ratio of the two largely prevailing components. These spectra can be theoretically reconstructed by four main components, which were assigned, in a progressively increasing order of binding energy (BE), to the residual graphene network (284.8 eV) and to hydroxyl (∼286 eV), epoxy (∼287 eV), and carbonyl (∼288.8 eV) functional groups. The success of the reduction process of GO samples with ascorbic acid and its extent are shown by the large change in the C 1s line shapes of the rGO samples (Figure 6b,d,e). The reduction process sets completely different atomic ratios among the C 1s peak components, with the epoxy-related peak undergoing the largest diminishing in the ensemble of functional groups. More quantitatively, the C/O ratios change in the following sequence: electrodic powder, from 2.1 to 7.2; commercial graphite, from 2.1 to 5.1; leaching, from 2.3 to 6.2. The values found are consistent with the range usually reported,^36^ while rGO values can be much higher in highly reduced GO. These line shapes have been theoretically reconstructed with the four main components discussed above for GO samples, plus an additional, slightly asymmetric peak at 284.5 eV, representing a partial recovery of the hexagonal sp^2^ carbon network.^37−39^ As can be inferred from Figure 5, this last component is the relatively most intense in the series in the case of the samples coming from electrodic powder (leached graphite and electrodic powder), which evidences the effectiveness of such treatment when graphite undergoes the lithiation/delithiation process in battery cycling. A qualitative look on the productivity rate of rGO obtained starting from the different graphite can be derived by the amount of rGO that remains in suspensions after 24 h (Figure 6b,d,f, inset). The rGO suspension derived from pristine commercial graphite did not display suspended rGO particles after 24 h, while, in contrast, rGO suspensions related to leached graphite and electrodic powder are both characterized by dark rGO suspensions. The higher graphene productivity resulting from the application of the Hummers’ method to the leached graphite and electrodic powder can be attributed to two main reasons. (i) The graphite lattice expansion induced by lithiation/delithiation during battery cycling weakened the bonding between graphite layers, leading to higher exfoliation efficiency.^40^ (ii) In addition, as proven by our recently published work,^26^ the graphite that derives from end-of-life batteries is preoxidized characterized by functional groups containing oxygen, which may facilitate the exfoliation.^40^ Preliminary cycling tests on the obtained rGO demonstrate its applicability as an anodic active material, delivering very good Coulombic efficiency at high current density (Figure S9).

## Conclusion

Starting from real waste EoL LIBs, we directly resynthesize new cathode material with low Co content based on layered lithium-manganese-rich material and rGO. The proposed recycling strategies involve a modified Hummers’ method that was applied on the waste electrodic powder obtained after mechanical pretreatment of mixed Li-ion batteries on a pilot scale. The modified Hummers’ method has allowed for the quantitative extraction of metals from the electrodic powder and the obtainment of graphite oxide without any metal impurities. The resulting solution contains the metals that constitute the cathode materials and, additionally, a huge amount of manganese used in the Hummers’ method. Remarkably, we found improved electrochemical performances for the recovered LMR if compared with the same materials without any doping, coating, or blending. We found silicate, sulfate, and Cu^2+^ impurities in the recovered materials. These impurities are currently employed to enhance LMR cathode performances in terms of both capacity retention and voltage fading. These impurities, in our case, lead to a high capacity retention of 73% after 100 cycles and a voltage decay per cycle of only 4 mV for a LMR material. In addition, we demonstrated that the rGO productivity increases when the Hummers method is applied on a graphite that undergone the lithiation/delithiation process during the battery life cycle.

## Supporting Information Available

Metal extraction rates using the Hummers’ method and conventional acid-reductive leaching; EDX and SEM images, cyclic voltammetry, and Co 2p, Ni 2p, Mn 2p, and O 1s XPS spectra of LMR; SEM images, Raman data elaboration, and rate capability test of rGO (PDF)
