# Supplementary material for: Upcycling Real Waste Mixed Lithium-Ion Batteries by Simultaneous Production of rGO and Lithium-Manganese-Rich Cathode Material
Source: ACS Sustain Chem Eng. 2021 Sep 24;9(39):13303–11. doi: 10.1021/acssuschemeng.1c04690 (PMC8493547; doi:10.1021/acssuschemeng.1c04690)
Supplement: Supplementary file 1 — sc1c04690_si_001.pdf [file sc1c04690_si_001.pdf]

Supporting information file

Upcycling Real Waste Mixed Lithium-Ion Batteries by Simultaneous Production of rGO and Lithium- Manganese-Rich Cathode Material

Pier Giorgio Schiavi*, Robertino Zanoni*, Mario Branchi, Camilla Marcucci, Corrado Zamparelli, Pietro Altimari, Maria Assunta Navarra and Francesca Pagnanelli

Department of Chemistry, Sapienza University of Rome, Piazzale Aldo Moro n.5, 00185, Rome, Italy

**correspondence concerning this article should be addressed to:*

piergiorgio.schiavi@uniroma1.it

robertino.zanoni@uniroma1.it

Number of pages: 6

Number of figures: 9

Number of tables: 2

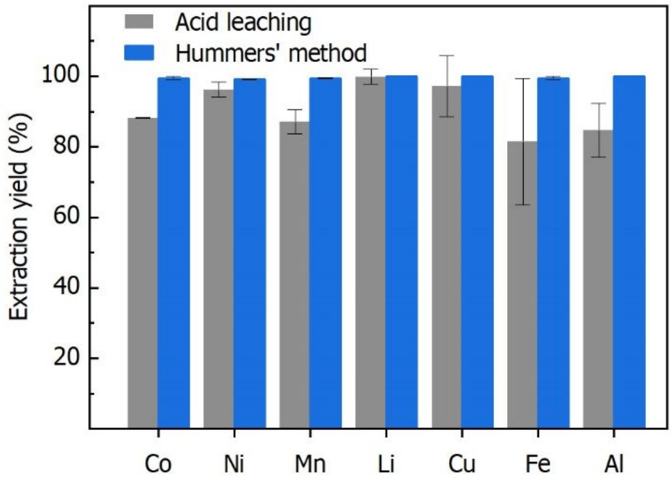

Figure S1. Metal extraction yields attained by performing conventional acid leaching and Hummer's method on electrode powder obtained after Eocrushing and sieving

Table S1. EDX quantitative analysis result on recovered LMR

Element	atom. [at.%]	error [wt.%]
**O**	72.99	5.76
**Mn**	15.83	1.31
**C**	3.29	0.38
**Ni**	3.62	0.48
**Co**	2.55	0.33
**Si**	1.51	0.11
**S**	0.17	0.04

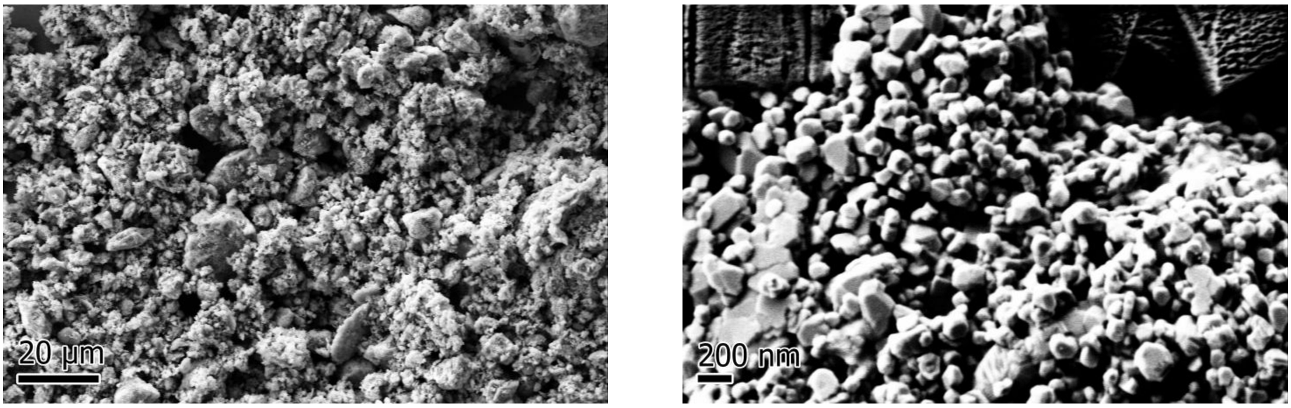

Figure S2: SEM images of recovered LMR at different magnitude

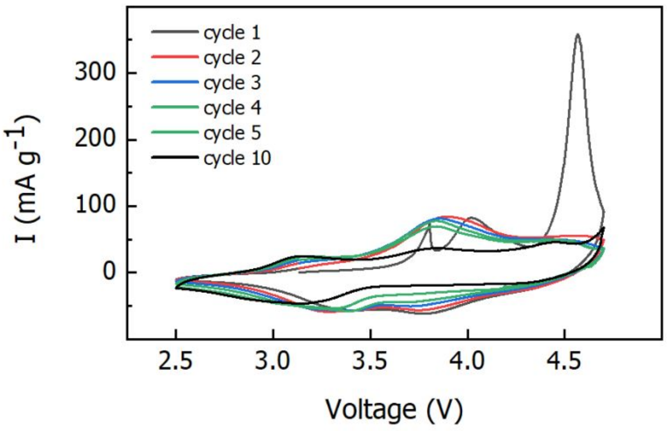

Figure S3: Cyclic voltammetry of LMR between 2.5-4.7 V and with scan rate of 0.1mV s^{-1}

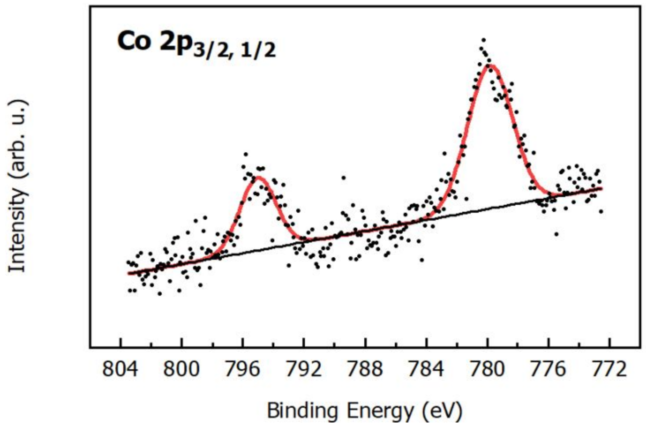

Figure S4: Co 2p spectra of recovered LMR

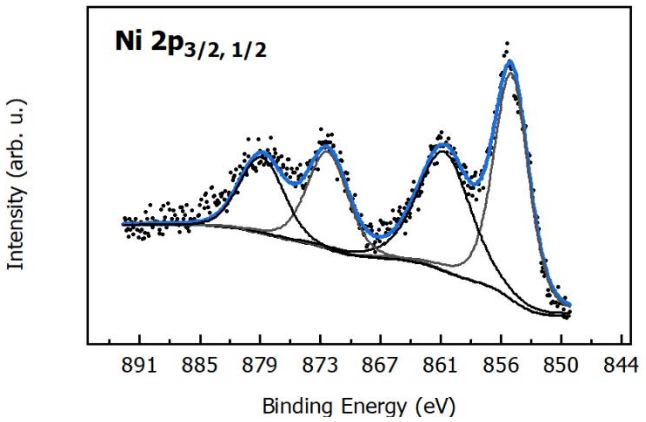

Figure S5: Ni 2p spectra of recovered LMR

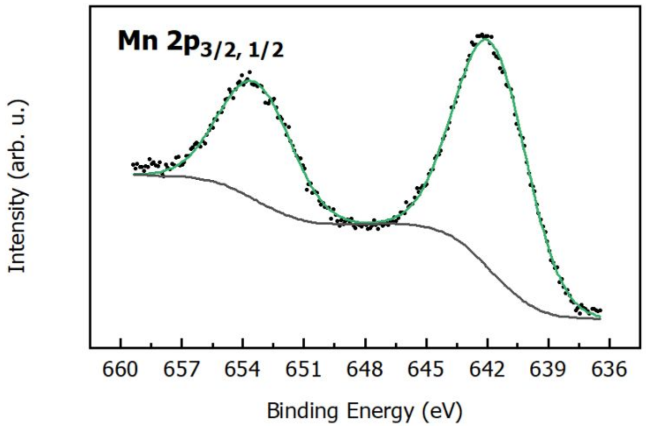

Figure S6: Mn 2p spectra of recovered LMR

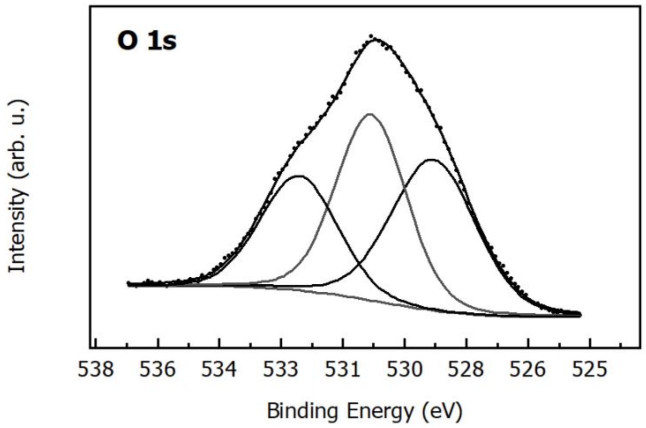

Figure S7: O 1s spectra of recovered LMR

Table S2: The lattice parameters of the LMR

**Sample**	**a [Å]**	**c [Å]**	**c/a ratio**	**error** [wt.%]
LMR	2.8549	14.2485	4.9909	5.76

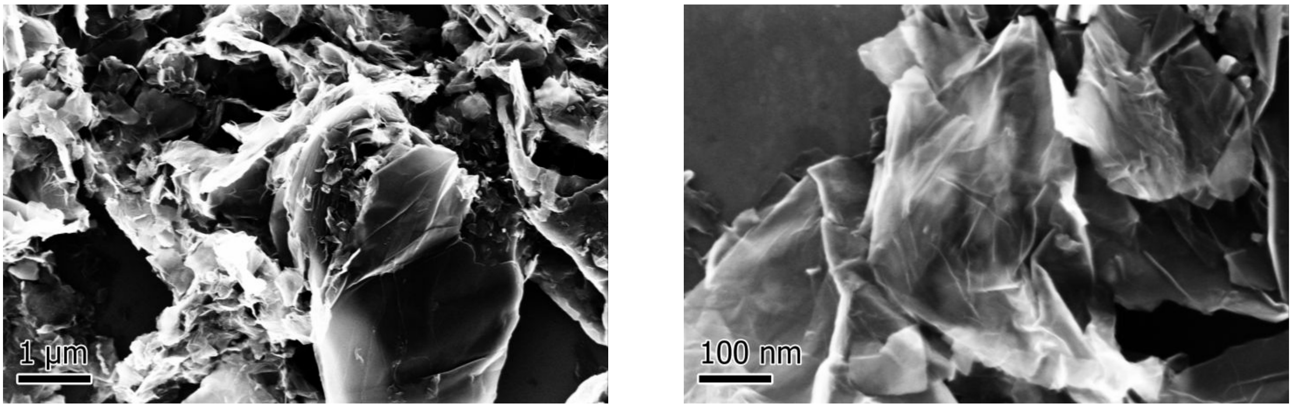

Figure S8: SEM images of recovered rGO at different magnitude

Table S3: Table S3: Values of Raman shift (cm^{-1}), ID/IG ratio and La (nm) for the reported samples. Full width at half peak maximum (cm^{-1}) of each peak is reported between brackets. The values of La have been obtained from the Eq.3 [1]

**Sample**	**D** [cm^{-1}] (FWHM)	**G** [cm^{-1}]	**D′** [cm^{-1}]	**2D** [cm^{-1}]	**D+G** [cm^{-1}]	I_D/I_G height	L_a (nm)
GO graphite	1351 (162)	1591 (93)	-	2699 (200)	2941 (210)	0.86	11.7
rGO graphite	1351 (96)	1577 (55)	1607 (45)	2692 (100)	2945 (150)	0.79	10.7
GO electrode powder	1352 (114)	1600 (95)		2722 (152)	2943 (149)	0.77	10.5
rGO electrode powder	1350 (96)	1574 (57)	1601 (50)	2691 (135)	2934 (160)	0.76	10.3
GO leached graphite	1349 (160)	1595 (91)	-	2687 (220)	2932 (206)	0.85	11.6
rGO leached graphite	1348 (86)	1572 (49)	1597 (50)	2688 (130)	2933 (151)	0.97	13.2

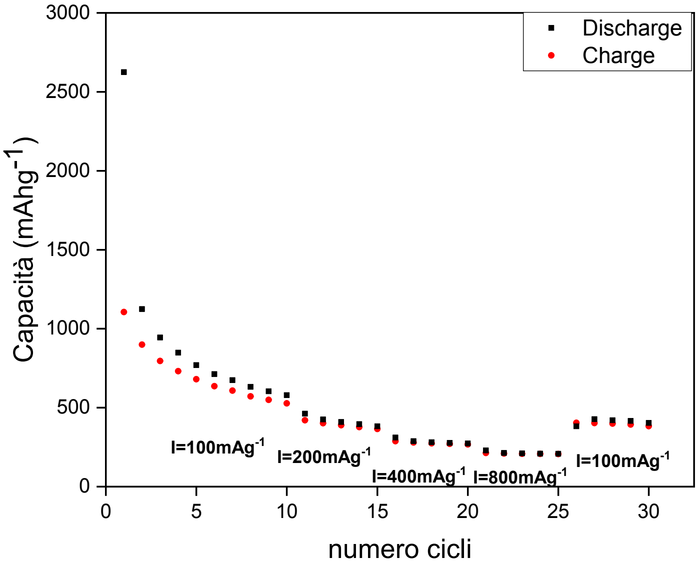

Figure S9: Rate capability performances of rGO produced directly applying the Hummer's method to the electrode powder. Potential rage within 0.02 -2.8V
